# Football Fandom as a Platform for Digital Health Promotion and Behaviour Change: A Mobile App Case Study

**DOI:** 10.3390/ijerph19148417

**Published:** 2022-07-09

**Authors:** Alex Fenton, Anna Mary Cooper-Ryan, Mariann (Maz) Hardey, Wasim Ahmed

**Affiliations:** 1 Business School, University of Chester, Chester CH1 4BJ, UK; a.fenton@chester.ac.uk; 2 School of Health and Society, University of Salford, Salford M5 4WT, UK; a.m.cooper-ryan@salford.ac.uk; 3 Business School, Durham University, Durham DH1 3LB, UK; mariann.hardey@durham.ac.uk; 4 Stirling Management School, University of Stirling, Stirling FK9 4LA, UK

**Keywords:** fandom, gamification, football, mHealth, mobile apps, digital, health, promotion

## Abstract

**Background:** The last decade has seen a dramatic shift toward the study of fitness surveillance, thanks in part to the emergence of mobile health (mHealth) apps that allow users to track their health through a variety of data-driven insights. This study examines the adoption trends and community mediation of the mobile fitness application ‘FanFit’, a platform aimed at promoting physical activity among sports fans by creating a fitness app branded to their favourite team for health promotion. **Objective:** Our study looked at the impact of a specially designed mobile app (FanFit) as a digital health intervention for initiating and maintaining physical activity as part of football club membership. Our analysis indicates that app users will adopt healthier behaviours as a result of the app’s sense of fan community and behaviour change. **Methods:** The findings reported here are based on an implementation of the FanFit app and, in particular, on those who participated in a more in-depth study (*n* = 30). These participants were Rangers FC supporters with a mix of genders *(n* = 19 males and *n* = 11 females). Focus groups and interviews were conducted with participants to ascertain users’ perspectives on the most effective methods for nudging users toward adopting and maintaining a pattern of fitness behaviours. **Results:** The findings show that the user community was interested in fitness and wanted to live a ‘healthy lifestyle,’ which was augmented and fuelled by the app’s competitive architecture design. Furthermore, the data reveal a new fan-health discourse about a person’s developing wants, talents, and identities as embodied beings. **Conclusions:** We have developed and presented valid links between the use of sports club apps and health programmes. The app could be useful for sports programmes and club providers looking for mHealth applications that provide community support through fan discourse with opportunities for both male and female fans.

## 1. Introduction

Over the last decade, there has been a rapid shift toward the study of fitness surveillance, aided in part by the proliferation of mobile health apps that allow users to track their health via a series of data-driven insights [1]. The creation of these digital health technologies is based on a technotopian vision of how data can positively transform societies by preventing health crises and giving citizens control over their health and care [2,3].

Prospective studies attempting to establish a causal relationship between physical activity and smartphone apps show that individuals who are more physically active and have a higher awareness of their health are more likely to meet the recommendations for both aerobic and muscle-strengthening activities [4], along with reduced stress and improved mental health [5]. These studies demonstrate how app-based interventions are tailored to motivational factors and how unhealthy behaviours (e.g., lack of physical activity) can be altered. While the analysed health benefits of increased physical activity may not accurately reflect the overall health of app users, especially those with clinically diagnosed health issues, the research demonstrates the potential for app-based interventions to generate new health outcomes. In this context, it is important to consider the relationship between health-app intervention, mHealth literacy, and the level of health-app use around changing health needs.

This paper focuses on FanFit, a customisable fitness app that can be rebranded. In this study, it was customised and rebranded for Glasgow Rangers Football Club fans and was given the name “FitBears”. The app concept was developed by one of the authors [6,7]. The FanFit app is a vehicle for investigating the expanding role of self-tracking data in shaping individual health-related behaviours, knowledge, and interpersonal relationships. The paper frames smartphone app interventions as an extension of self-tracking surveillance and enhanced physical activity [8,9,10,11], in which real-world fitness activities and app emotional affordances interact with discursive forms of fitness anchored in each user’s allegiance to their sports club.

By focusing on individuals’ changing needs, capabilities, and identities as embodied subjects in self-tracking fan-based digital communities, the current study identifies a new fan-health discourse. Previous research [12] has highlighted the role of social media fans in adolescents’ acceptance of wearable health trackers as part of a physical activity intervention programme. Other research [13] has found similar fan dimensions in a systematic review of SNS-based interventions to support young people’s mental health and the importance of online peer-to-peer support.

The project’s goal was to improve football fans’ understanding of digital fitness, gamification and socialised aspects through competition and smartphone fitness use while promoting healthy lifestyles. A customised version of the FanFit app called “FitBears” was created for Glasgow Rangers Football Club (Rangers) fans in Glasgow, Scotland, and was named after the official Rangers Mascot, Broxi Bear. The project was a result of a collaboration between the University of Salford and the Rangers Charity Foundation (RCF). The FitBears app was released in November 2019. It brought the Rangers and RCF brands together, as well as information; social media feeds, fitness features and fitness leagues.

**Figure 1:**
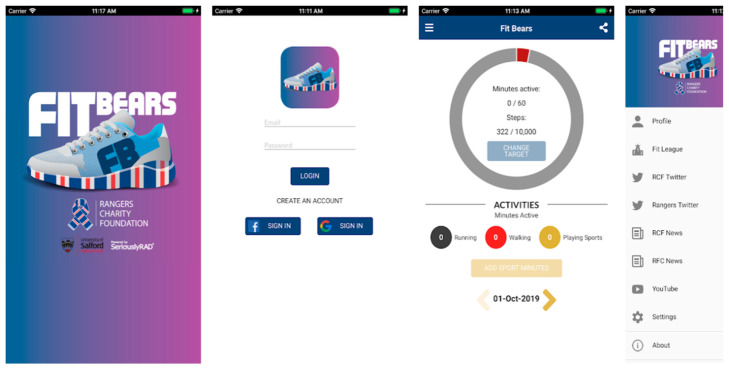
Screenshot of the FitBears Smartphone App.

FitBears, which is powered by FanFit software (version number 1.3.6, developed bespoke for the University of Salford, Salford, UK), was designed to work with only an Android or iPhone built-in step tracking but can also integrate with a variety of fitness wristbands. It presented users with data on walking and running in a simple, fun, engaging, and visual format. Fans could compete for digital badges and physical prizes by logging their steps in monthly, global, or private leagues. As a result, the app combined fandom, club engagement, social capital, and community participation to reach people who might be more difficult to reach and less likely to engage in more traditional lifestyle interventions. Figure 1 provides a number of screenshots from the app. 

The FitBears app was designed to read existing news and social media updates from both RCF and Rangers. Pooling multiple feeds and updates into an app is fairly standard for a sports club app, but there are many sports clubs that do not have any official app. Sports clubs face many barriers to innovation, including time, money and expertise. In addition to this central pool of club information, news and fixtures, FitBears was designed to track walking and running activity through different kinds of fitness wristbands or just through a standard smartphone using Google Fit or Apple Health, respectively. This enabled global, monthly or private leagues to be created. The advantage of this is that, unlike a standard fitness app (such as Fitbit), fans could use different kinds of fitness bands to compete with each other, or no band at all. The private leagues were particularly well-reviewed because fans could start these with their friends and fellow fans. It had the ability for fans to set up and share the league with a sharing code. Furthermore, automated and custom push alerts were used to remind fans to use the app, report on steps and promote other relevant events at the club.

This version of the app did not target mental health aspects specifically but physical aspects through the counting of steps and competition. However, fans did request a chat function that would sit behind the private leagues, so whilst the private leagues did create a friendly competition, fans were keen to take this a step further to build more of a sense of community.

Subsequent ‘white label’ versions of the app have been produced for other organisations, such as CITe football as medicine. White label refers to the ability to change the branding of the app, which is important to provide a brand that people can identify with. This is one key differential of the project in order to build a sense of personalisation and identity. For more information about the app itself and subsequent versions, please see www.FanFit.co.uk (accessed on 1 July 2022).

Within the first six months of the project, from its launch on 3 November 2019 to 3 June 2020, according to Google Analytics for the app, there were 7200 fans that used FitBears. They spent an average of 9 min and 13 s using the app. A total of 79.5% of the users were recorded as male and 20.5% as female. The majority, 6.9 K, were from the UK (top cities Glasgow, London and Edinburgh). The group of 35–44-year-olds was in the highest bracket in terms of age group (385) (See Appendix A).

The findings presented here are based on a larger FitBears study, but the focus of this paper was on working closely with a smaller group (*n* = 21) of Rangers fans who used the app as part of a small study or as prize winners. The study was carried out in Glasgow, Scotland, which has a lower life expectancy than many other European cities, giving rise to the phrase “The Glasgow Effect” [14].

Heart disease is the leading cause of death in Scotland, with mortality rates that are significantly higher than in comparable cities. Because the disease is also associated with inactivity, the project was created to address this using brand association and fandom. FitBears marked the first time that many fans had actively tracked and visualised their fitness as part of a larger study.

## 2. Theoretical and Conceptual Framework: Fan-Health Discourse and Human–App Assemblage

A growing number of studies are being carried out to investigate the development and optimisation of health interventions aimed at football clubs, including players and fans [15,16]. Recent research on sports fan engagement on social media has clearly identified new modes of interaction and changes to the fan–athlete relationship in terms of cultivating a sense of belonging through fandom (MacPherson and Kerr, 2021), and most recently, engagement through digital teams considering attendance restrictions at football games due to COVID-19 [17]. Several studies have attempted to understand how predominantly male football fans may engage in weight loss programmes promoted by football clubs in this context [18,19]. Sociological accounts of football continue to emphasise how gendered identities influence fandom bonds and sports consumption, as well as how hegemonic masculinities are reproduced at these collective levels (even when female fans are present) [18]. In this context, football fandom is a bounded identification, and studies on global football fan behaviour show that supporting a football club is a life-long project that begins at a young age, is frequently passed down through male family networks, and ends with the fan’s life [20,21]. Given the rise of women’s football and the changing ratio of women supporting clubs, the gender gap in sports fans is significant in terms of the extent to which fandom forms part of an individual’s identity as well as differences in levels of motivation for sports consumption. Scholars [22] have emphasised the issue by describing how researchers appear to “add” women into analyses, presenting this as a convenient by-product of the main research. Researchers [23] have investigated the development of ‘Football Fitness’ programmes within sports studies, which has useful implications for this study concerning girls’ and women’s interests in sports and fitness.

Within the constraints of how the technologies are encoded and sports and fitness programmes are treated differently, the structural elements of smartphone health apps offer a variety of different health-tracking opportunities [24]. What we hope to accomplish with this paper is to add to the growing body of research on the lived experiences of self-tracking [25]. A major contribution of the study is to acknowledge the confluence of community and different gendered fitness practices of the users. This indicates that it is more crucial than ever before for us to integrate fandom and social identity into the context of everyday lives. During a period of rapid socio-technical change, a renewed emphasis on comprehensive descriptions of emerging digital health knowledge—prior to any rush of support for health behaviour change through smartphone applications—is a good approach. Before we can successfully position fandom and new digital objects of social identity within wider theoretical frameworks, we must first comprehend some of their fundamental qualities at the level of gender, health data gathering and analysis, and community connections.

Health knowledge is frequently associated with the assumption that male physiology is normative, with the result that women’s bodies are particularly vulnerable to being ignored [26]. More recently, at the heart of the human–app assemblage, such data are increasingly contributing to people’s concepts of selfhood, forming “numbered lives” [27] in ways that affect men and women differently. Relevant to this study is how, until recently, women were overlooked in sports fandom, particularly football fandom [22], and the synthesis of digital interventions that are typically used as default male physiology. In fan research, there are broader gendered issues that underpin much of the self-tracking software modelling on male and female users. Previous research [6] has discussed how professional sports clubs are attempting to engage female fans through digital communities to foster inclusive fan cultures. One of the study’s most significant contributions is to investigate how gendered fandom experiences differ in relation to the use and desire for an affiliated fitness tracking app and the way in which gender is a significant predictor of achieving health goals [28].

Deborah Lupton’s [11] extensive work on self-tracking and increased personalisation of health data via smartphone apps have been widely used in the study of health and critical examinations of human-app assemblage [29]. These studies highlight new relationships with health data and technologies that provide a more intimate experience of fitness, as people may never be able to disconnect from their data trackers, which use wearable devices to monitor their activity and sleep. According to Lupton [30], such relationships are examples of human–app assemblages, in which health knowledge constantly reconfigures lived experience, and users exist on the ‘data-gaze’ frontier, as envisioned by other scholars [31].

One of the things we notice in this paper is how self-tracking technologies act as incentives for the expansion of David Beer’s [31] data frontiers: ‘ushering in the expansion and intensification of data within organisational and social structures’. This reframing is important when health data frontiers are not simply contained within organisational and social lines; rather, these types of data analytics reflect emerging social activities and point to new territories in which health data may be used in the future [32,33]—for example, by community groups and, in this study, football clubs. This interpretation has far-reaching implications in areas where there is little understanding of personal health records and commercial data usage, as well as how users may be willing to invest in fitness apps if they are directly linked to offline community groups (e.g., football clubs). Our focus here is on some of the significant developments in fandom culture, and we are interested in trying to ascertain what social identity contexts are relevant to understanding the adoption and use of smartphone health apps. Recent studies suggest that the fast growth of smartphone health applications is rewriting data linkages, altering health professional designations, and rearranging caring relations inside the home. Moreover, these technologies have been rapidly adopted by users and incorporated into self-tracking practises within what are frequently viewed as mundane everyday activities; as a result, they are at risk of being dismissed as unimportant unless we remain vigilant to the broader significance of social identity in creating new forms of health behaviours—such as fandom.

With all of this in mind, our research focuses on three major themes. First, we explore how fitness apps are incorporated into the lives of football fans, as well as the barriers that fans face when it comes to engaging with self-tracking data. Second, we look at how fans’ connections with other fans enable and motivate the community. In the final theme, we identify aims to comprehend the interplay of gender and self-tracking data. This three-step process is intended to ensure that we accurately report the motivations that stem from fandom and the efforts of sports marketing initiatives to recruit more female consumers.

## 3. Methods

Ethics approval for this project was granted by the University of Salford ethics panel (SBSR1819-27). The focus groups and interviews were part of a larger study that ran over the course of 6 months and included several qualitative and quantitative methods (paper in draft). Overall, the project was carried out in several stages to ensure we could engage with users throughout the design, launch and testing of the FitBears app. Throughout this project, two of the authors worked closely with RCF, who supported the delivery, recruitment, and facilitation of the project.

### 3.1. Recruitment

Recruitment was assisted by RCF, who facilitated contact with fans and supported the running of competitions. Participants were initially recruited using purposeful sampling from Football Fans in Training (FFIT) [19]. The FFIT programme is designed to provide fans with an opportunity to work on fitness and health through programmes run with football teams around Europe. These fans were therefore engaged in wanting to make changes to their fitness and with the club.

Following this, with the support of RCF, participants were recruited for a small sub-study to further explore perceptions of the new FitBears app, as well as those who won FitBears competitions throughout the study period were invited to take part in winners’ interviews during the prize-giving event. Of those within the FFIT programme, some were known to each other before they took part, while the competition winners were not known to each other. All those who took part (*n* = 30) were Rangers FC fans, and a mix of genders was included (*n* = 19 males and *n* = 11 females), with all being over the age of 18.

### 3.2. Procedure

**Figure 2:**
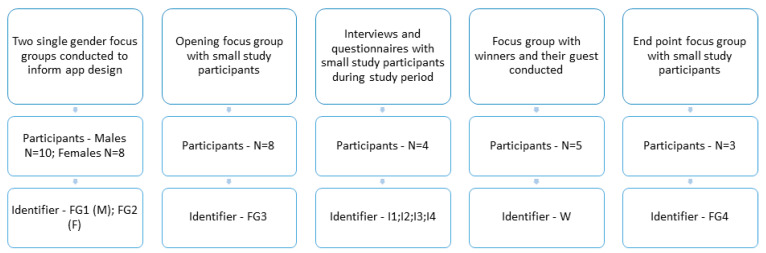
The process of data collection through the study period and the participant groups.

Events took place at the Rangers ground (Ibrox) or via telephone and lasted no more than one hour, with each being recorded for transcription purposes. The data reported in this paper were collected using a combination of face-to-face focus groups and individual phone interviews (as set out in Figure 2). The initial two focus groups informed the remainder of the study, with questions for later stages drawing on the outcomes of this and a larger survey/social media engagement with app users (reported elsewhere). Using a variety of methods allowed us to remain in contact with those in the smaller study (through phone interviews part way through and weekly online questionnaires).

Those taking part in the focus groups and phone interviews were part of the Rangers FFIT programme, whereas those who took part in the small group winners’ interview were from the wider Rangers supporting community and were the prize winners.

Initially, two single-gender focus groups (*n* = 8) were conducted with FFIT participants to understand how they use and view self-tracking apps, features which are barriers and facilitators, their current health and wellbeing behaviours and their initial view of the new app being developed. These two focus groups were conducted by gender due to how they attended the FFIT sessions; however, this highlighted how gender was an important part of the findings and would be in other parts of the research.

The second set of data was generated through a small sub-study that recruited n = 8 participants (*n* = 4 males and *n* = 3 females) at different stages of the FFIT programme. This was designed to provide longer-term and more in-depth insights into the experience of using the app and any behaviour changes that occurred over an 8-week study period. Participants were asked to use the app, along with a provided Garmin Vivofit wristband for the study period. A focus group was conducted at the start to collect initial thoughts on the app, self-tracking, health and wellbeing. During the study, feedback on their experience was gained through an online questionnaire and phone interviews. Then, at the end of the study, the participants were asked to again take part in a focus group, although not all were able to attend. All those who took part in the initial focus groups and the small sub-study were over the age of 18.

Finally, throughout the study, monthly competitions were run: firstly, two competitions for the most steps and, after this, random draws from those who sustained at least 7000 steps a day. The project team decided 7000 would be achievable for most fans. The winners and those accompanying them to the winner’s event were recruited to take part in a small group winner interview (*n* = 5) to understand how the app was used, behaviour changes that occurred and any barriers and facilitators.

### 3.3. Analysis

**Figure 3:**
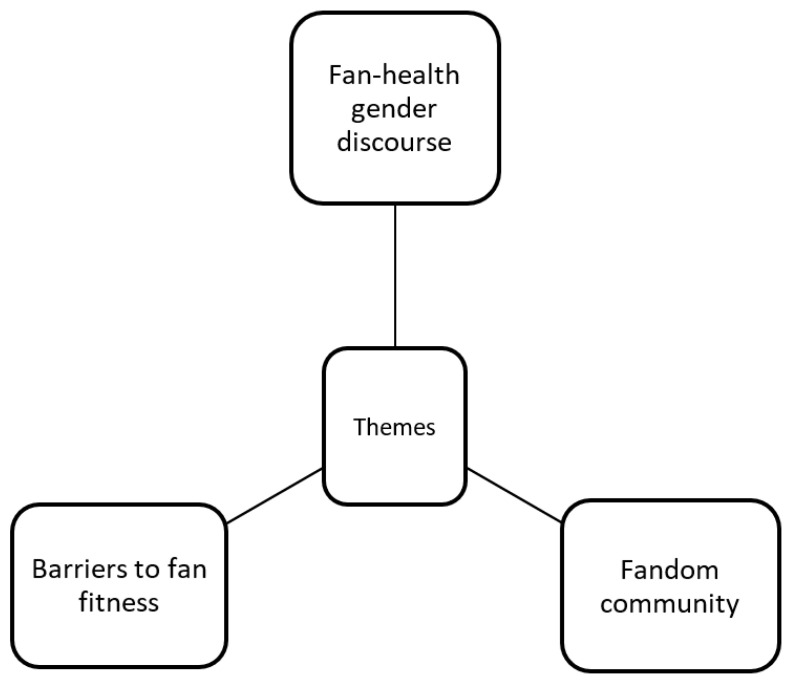
Overview of the three core themes to emerge.

Interviews and focus groups were transcribed by an external transcription company (OutSec) and then checked by the researchers for accuracy. We used a thematic approach to analyse the data aligned with the work of Clarke and Braun [34]. Using NVIVO-12 (NVivo), we initially generated *n* = 17 codes using an inductive approach. NVivo visualisations were created, including a Sunburst chart of codes, which was used in conjunction with the literature review and the original research question to create themes [35]. In conjunction with the literature review, as shown in Figure 3, the three core themes were generated: (1) Fan-health gender discourse, (2) fandom community, and (3) barriers to fan fitness [34].

## 4. Results

### 4.1. Fan-Health Gender Discourse

The body/self as it is enacted through self-tracking apps is both the product of health data and open to interpretation. Health data tracking is far from neutral and reinforces ‘highly reductive and normative’ ideas of what is ‘good performance’ [30]. In this study, the comparison of achievements by sex also reinforces gender stereotypes both in terms of fitness levels, willingness to compete and focus on performance. Part of the differentiation reflects the heavy use of the app by male users, conceivably occurring due to the Rangers FC fanbase.

In the period of study, FitBears app analytics showed that 79.5% of the app users were male, and 20.5% were female (see Appendix A). This is in line with data that show that currently, there is still a predominantly male fanbase for football clubs such as Rangers and a divide in which sports are taken up by different genders. For instance, previous research [36] has found that sports participation rates in Scotland are significantly higher for men; however, more women participate in recreational walking (an activity that is a key feature of the app). Part of the design of the study was to capture gender differences, involving single-sex and mixed groups where participants talked about their experiences with the app.

In the female group, there was a significant experience of using other fitness apps. When asked about features, they reported that food and calorie intake was a priority:

*… is there a space for your food intake, how many glasses of water you’re drinking, things like that? I know there is an app that you can download to go, ‘It’s nine o’clock, drink a glass of water’. Something like that, I think, would be good to add to the app, to get it all together in the one thing.*

(FG2, Gladys)

Such features are not shared with other users in the same way as, for example, the number of steps in a day, and do not appear on ranked leadership boards. For the women users, these hidden data hold significant value, providing better knowledge about their diet and using these data to control desired changes in eating habits and subsequent health impacts.

Both being able to extend fitness data to incorporate nutritional data and following friends extend the context of self-surveillance as part of the quest for improvements in personal health and, at times, being invisible to other fans. Here, the quantification of supposed neutral app data is valued over their own embodied knowledge of their bodies [26,30]. This reflects the rhetoric used to promote commercial apps and in the promotion material of apps that suggests ways users (typically women) can achieve a better level of knowledge about their bodies by recording signs and symptoms and using data science to achieve health-orientated goals.

For the female users of the FanFit app, self-esteem and self-improvement were supported through community motivation through private fan leagues. It was found that for many in the female focus group, having integrated features, rather than the need for a third-party app, was felt to allow greater social interaction (e.g., a chat element or highlighting a friend’s data easily) and was seen as something that developed a core sense of self-esteem and support:

*“[…] so you can find your friends and add them, but you can only see their improvements in terms of fitness”*

*“Because that way, you can talk about your challenges”*

(FG2, Karen)

In a similar vein, a social perspective was evident, albeit with a different set of emphasis, within the male focus group, who also felt there would be a benefit to being able to communicate with others within the app:

*“It would be creating a bit of competition you know, and I would say from a banter point of view, ‘ah you’ve done nothing this week’, you know.”*

(FG1, Frank)

*“I think with the challenges because you’re constantly wanting to beat your last challenge. And if you’re doing it with your friends, then you’re like, ‘No, she’s …’ [over talking] It would be about motivation.”*

(FG2, Karen)

Self-tracking helped users to feel more in control, but in this study, however, there are clearly different expectations about the app’s primary use between the female and male participants. In the male focus group, one participant characterises his investment self-tracking as ‘very obsessive’:

*“I find it [self-tracking] very obsessive, yeah, I think because you tend to watch it and think oh, I’m getting near my target and you kind of get, I kind of get agitated”*

(FG1, Ben)

Similarly, the app amplified this kind of obsession both explicitly (engaging in challenges, having leader boards to enhance competition) and implicitly (through communications and comparing data with other users). Like the female users, the male participants commented on their sense of enjoyment from the competition and being challenged, which was viewed as self-motivating. Speculation about the impact of using competitive elements within the app centred on the elements of ‘friendly competition’:

*“I think from a fit point of view, I think it would be quite interesting […] obviously there’s a lot of banter you know. Aye, there’s some non-athletic [laughter]. But I think from that point of view it would be creating a bit of competition […] from a banter point of view, ‘ah, you’ve done nothing this week’, you know.”*

(FGM, Malcolm)

Likewise, some of the female participants were also using the private leagues in the app and the competition as motivation [37]:

*I saw him last night and I am second, but I am only a few thousand behind him, and I thought, ‘This week I am going to work hard, I am getting back to first place’. And the two of us have a great camaraderie, to say, ‘Right okay, I will be watching what you’re doing next week’*

(Phone interview, Sarah)

As demonstrated above, there are growing entanglements between the data-sharing practises of self-tracking, camaraderie, and the desire to engage in ‘banter’ and social interactions. These fans felt their experiences were enhanced by activities that added to the visibility of their personal data. Rather than express a desire to keep data tracking hidden, the sharing of data publicly enriched the experience, generating experiences of competition and social interactions. These elements underscore the importance of the social aspects of the app, albeit in very different ways.

Further, the entanglement of data and socialising reinforces the significance of self-surveillance as social life and selfhood combined within the app to engage user interest and maintain participation. In this way, inscribing a competitive value to data tracking (whether hidden or visible) attaches new meanings to health data that carries a ‘social value’ relevant to group activities, which ‘renders them all the more necessary for the monitoring of one’s physical performance’, while also underscoring the playful competitive environment of the app.

Where the production of quantified data enabled users to form their subjective experiences of the app, the production of further opportunities for competition contributed to an overall sense of competition for competition’s sake rather than health gains. In the later mixed focus groups, there were further observations regarding the hyper-competitiveness of men with FFIT and fitness apps, a female participant noted the ‘terrible’ competitiveness of men:

*“The men are more competitive on this course, believe me, they are worse than women, the men are terrible. They are absolutely shocking, honestly”*

(FG2, Kelly)

*“That’s right, but they are more competitive. It’s like, ‘Oh, he’s lost a half-pound more’ … men are worse than women for that.”*

(FG2, Sarah)

However, a male participant believed there were many competitive women, but emphasised this added motivation to try and be fitter or do more steps:

*“I think some of the ladies are very competitive, from the sound of it, you know? So, we need to beat them.”*

(FG1, Gareth)

Overall, it seems that FanFit users are motivated to engage with self-tracking data in different ways from personal targets, rivalry, prizes, or step league positions. Personal targets set by themselves to beat each day and then, furthermore, trying to get more steps recorded to compete with their friends, or at least not to slip down the league table and be considered lazy or that there may be a problem. To return to Ben’s comment, an ‘obsession’ with competition was clearly influential in achieving certain fitness levels. Another participant reported that these ‘obsessions’ would sometimes manifest in unusual behaviours such as jogging on the spot or marching around the house to achieve more steps and win a league:

*“I was jogging on the spot for at least two hours in the evening. I was getting up at 4.00 am, and I was jogging on the spot while the kettle was on and getting breakfast.”*

(WI, Phil)

In anticipation of comparing health data, the progress of one’s fitness levels was dominant in understanding fitness outcomes and app use differences between the sexes. Gary’s comment ‘*so we need to beat them*’ clearly articulates the desire to retain control of his body in comparison to his perception of how her observed female fans use the same app. The way the male participants were keen to be seen as visibly competitive correlated with two interrelated desires: first, to be seen performing within the app; and second, to build up a competitive reputation based on this performance.

Both desires reflect Esmonde’s [38] observations of gendered self-surveillance and tracking practices. Competition through the FanFit app reinforced the devotion (much like fan loyalty) to fitness and how people use tracking and competition differently. Both the men and women taking part were active and capable in making decisions about their health and fitness, but invested time in using the app in different ways to achieve different social outcomes (e.g., to connect with others through the leagues, the competitions or through using it to support goal achievement).

### 4.2. Fandom Community

Self-tracking can assist in the dismantling of geographical barriers, and users benefit from a digital community that is not bound by locations or nations but rather constructed by fandom [13]. For example, bridging the gap between Germany and Scotland:

*“I enjoy using the app since I live abroad (Germany) and it gives me another way of staying in touch with the club whilst staying active, something which has become really important to me in the last few years. Using people’s passion for their football club as extra motivation to stay active is a great idea.”*

(Email, Janet)

There was also evidence that the community was being enabled to take care of others; for example, in one private league, a member had unusually slipped down the table, giving others cause for concern:

*“it gives you means of saying ‘I wonder if they’re alright’. I contacted [him] and I found out he’d had an accident; he’d fallen down the stairs in the house. So that’s why he been laid up for a while.”*

(FG4, Robert)

The community also produced a fun aspect and banter that were often emphasised as support to motivate each other when a participant was doing less steps than usual (*flagging a wee bit*),

*“But likewise, when I see him flagging a wee bit I’ll say to him ‘What’s up? What you doing? Where have you been? Get out the pub’.*

(FG4, Robert)

The motivation to continue tracking with other fans therefore extends fitness beyond the self to fans entrenched in connection with others and ways to support one another.

*“But I think, like you were saying, talk to each other, we can say, ‘Does anyone want to go for a run today, or a walk, who’s up for it?’ You know? And just organise something like that in the group you’re in, I think that would be good.*

(FG2, Sharon)

The fan relationships demonstrate the physical connections to motivating people, which can occur more in smaller groups (e.g., private leagues) (Although participants are often looking out for and encouraging each other, the concept of competition between each other was seen as a key motivator:

*“because it’s that wee bit of competitiveness, you know? I don’t know if you’ve got last month’s figures there, Sarah and I were quite close together and again this month, so there’s that wee bit of competitiveness between certain members and other people”*

(PI1, Colin)

*“So, I saw him last night and I am second [in the app private step league], but I am only a few thousand [steps] behind him, and I thought, ‘This week I am going to work hard, I am getting back to first place’. And the two of us have a great camaraderie, to say, ‘Right okay, I will be watching what you’re doing next week’. So, that’s just a wee private league, which has been really good fun, it’s been good.”*

(PI3, Sarah)

Here, some affinity is born out of their common fandom, reinforcing their sense of belonging and community: tying both to the club and to other fans. These relationships created both competition and camaraderie.

These examples highlight how self-tracking technologies blur the spatial boundaries between public and private fan relationships; essentially, FanFit/FitBears users are bringing self-tracking into their private/intimate spaces and creating new forms of community and social capital [6]. As a result, users are not only constructing new methods through which they are personally responsible for their own health but are part of a network of actants, which include the various software algorithms employed along with other users and discourses around ‘fitness’ with those users [32,33]. Here, we can underscore Lupton’s human–app assemblages to explain the interrelationship of the app (technology), data produced, users/bodies recorded, and shifting fitness and social activities [11]. In this regard, we are acknowledging the role played by the app and data (the non-human aspects) in producing knowledge about fitness and health and subsequently supporting changes in behaviour.

### 4.3. Barriers to Fan Fitness

There are strong motivations to engage with and experience self-tracking apps [11]. However, while these studies account for the strength of motivation as being a primary incentive, less is known about the barriers users overcome when they first start engaging with self-tracking. We address this gap through the FanFit implementation of FitBears and associated research. As fans started using and engaging with the app as part of the study, it could be seen that there were several initial barriers. The first barrier identified related to participants finding limitations in the data captured:

*“I’ve bought a bike as well, so I was doing my walking, and then I got a bike. On a bike it doesn’t count toward your steps”.*

(WI, Charlie)

Charlie felt he was putting in the effort to cycle, and therefore, the app should also record and reward him for that activity by recording that data, and furthermore, exclusions in data tracking represent a clear barrier to the enjoyment of the app, and anxieties were expressed by participants who worried about ‘missing’ data and moments when their activities would not be tracked.

The research team received a number of messages on social media and emails from fans that had lost step data for some reason or asked if the app worked with particular brands of wristbands, for example—‘*Will the app work with my FitBit?*’ Three main reasons for issues being reported were identified: technical faults occurring, undertaking a non-step counting sport (e.g., cycling) or using an incompatible fitness wristband without a connecting app such as Google Fit.

In terms of the accessibility of the app for different user groups and how tracking cycling data may be linked to this, one participant highlighted that it “*Maybe help for wheelchair users and the like.”* (WI, Charlie). This is also significant because this participant is not only thinking about their own cycling but considering barriers to engagement by people who are not able to record walking steps, something which is often not accounted for in fitness apps.

Other participants noted their use of self-tracking data by articulating this in numbers such as league position or number of steps while wanting to understand if the research team had sight of these data:

*“I don’t know if you’ve got the figures there, but in December I started off doing roughly 18–20,000, and then around about the 4th or 5th … so, about the 14th I was lucky if I was doing 13,000 a day.”*

(PI1, Calum)

This comment also relates to how our users were discovering self-knowledge through the number of steps, but also an awareness that within many health apps, there are surveillance elements where others (e.g., the research team) can view data if they choose to at individual or more often aggregated levels. In this study, however, awareness of being observed by a third party was not found to be a major barrier to engagement, which aligns with the findings of other studies, such as Lupton [11], which found that participants had little or no concern about how their health data are used.

A more significant participation barrier for some to engage in physical activity or activity to improve this was individual health issues, a challenge that many interventions must consider.

*“Because obviously I had a gout attack at that point, and I was struggling to walk on it. So, that’s the only thing that really stops me.”*

(PI1, Calum)

Within this app, users are able to set personal goals, meaning that they can tailor their steps to their ability, something that can improve the accessibility for those with health issues that can limit their ability, e.g., could they move from a few hundred to a thousand steps a day, which for some people would be a big shift in their activity. Linked to this, when discussing competing in the leagues and a personal target, it can be seen that a negative health status or having an accident were considered to be significant barriers by some participants. Equivalent concerns have been found related to diet tracking apps [39],

*“Well, I’m trying to…my aim is still to try and get my 60 min of exercise in a day which I’ve not been managing because I had quite a significant accident, so I wasn’t able to for a while but I’m now beginning to get that back up.”*

(PI4, Doris)

This is where it is important for those delivering interventions or trying to support people with their health to have ways that all can feel included, e.g., competitions for winners and those who sustain a more manageable target or even those who maintain a level that could be much lower than some, but they do it consistently over a month. This can promote inclusivity and encourage motivation. For many participants, time was a significant determinant of levels of physical activity. For example, family responsibilities, work and other distractions were contributing to challenges with engaging with self-tracking data.

*“I can drive nine hours a day, sitting in a truck driving somewhere and having to sit in the truck for a break, and then you’re just driving again. Maybe do it for a couple of minutes and then you’re back in again.”*

(FG3, Robert)

Jobs such as office work or driving that require a lot of sitting down were cited as a barrier to gaining as many steps through the app as they would like. Again, this could be where individual goal setting can support people feeling they can achieve their targets and improve over time. However, as scholars [40] have observed in the study of mHealth tools in the workplace, such tools allow for a systematic review of progress; however, this is put at risk where there is an over-emphasis on health evaluation, and employees feel obliged to use such tools.

These barriers prevented and impacted play with self-tracking data due to several different reasons, which were both personal and digitally derived. Scholars [31] have observed that personal data describe multiple ways in which data have become commodified and the methods by which health data have become a more comprehensive form of physical capital. For many scholars writing about self-tracking, a significant risk to self-tracking users is a period of ill health or an ‘accident’ that represents a real risk to physical capital, such as those described by Doris above. Furthermore, Sharon [41] describes the disempowering effects that greater health monitoring can have. Through the ordering of individuals into leagues and competitions created, there is a risk that inclusion and exclusion can have negative effects. Personal information gathered via the use of apps allows individuals to be grouped or sorted into discrete categories and classes based on this information and then subjected to assessments based on prior assumptions. In conjunction with some of the barriers to attainment highlighted, this poses further considerations for the polarised debate regarding empowering or disempowering individuals to engage with self-tracking data.

## 5. Discussion

This paper sought to enhance our understanding of how smartphones can be used as a vehicle for health improvement and evidence of how self-tracking apps can have a substantial influence on users’ experiences of health. By focusing on engaging a group of people who have a collective sense of motivation through their fandom for a football club, we highlight a new way to explore health surveillance. There are commercial aspects of app development and building on motivations linked to fandom and linked to self-tracking. This ties into the fact that health surveillance is increasingly market-driven, leading to different conditions of community relations and expectations around social connections within health apps.

Our study further highlights the role of the club and ‘fan’ status and new opportunities to help people overcome barriers to using fitness apps by engaging them through their club. The insights from these findings could also be extended to other modalities of health surveillance that are not necessarily fan-based but that attract people to a collective desire to engage with a brand, for example, the ‘cult’ status of other physical activity apps such as Zombies, Run! Our research also speaks to the continuation of taking health away from just being led by health experts to move it into the hands of consumers bringing together our observations about how self-tracking can be interpreted by the individual and other users.

By accounting for the relationship between fan behaviour and health activities, we improved our understanding of the context of fitness app use among Scottish football fans in this study. The promise of empowerment is critical to overcoming barriers to app use, while also raising interesting issues about reliance on others within fitness communities. In contrast to other studies on self-tracking, the relationship between football fans and clubs enhanced the appeal of self-surveillance, encouraging users in ways that stimulated interest in fitness and devotion to their club. In the design and implementation of interventions, we identified a clear set of values and expectations that can be linked to border socio-cultural transformations around health, fitness, and bodies. Furthermore, we demonstrated that there are various expectations in the app’s design regarding how other users will view personal data and how individuals seek to interact with others. There is a risk that apps such as FanFit compel users to view complex health phenomena and selfhood as data (sometimes with commercial interests at stake), and thus constrain alternative means of health support and self-expression [41].

As with all self-tracking apps, there is a challenge that they will not suit all those using them; however, working with an existing brand community, providing more than just fitness features in the app, and allowing a number of fitness bands to connect through one app (e.g., Garmin, FitBit or Withings), as well as providing motivational factors (e.g., competitions, leagues) has been shown to encourage a diverse range of people to monitor their physical activity. Despite this, we did find that there were several features those using the app would have liked to allow them to enhance the building of communities and connections, such as an inbuilt chat feature or closer interaction with famous players. This is interesting since the data reveal how users are looking for apps to be able to take on several roles from self-monitoring, communication, news, and support rather than users wanting to engage in a few apps to carry out these features.

## 6. Conclusions

This study’s data make two distinct contributions to the literature. First, the study demonstrates that, even when ‘Fandom’ is considered as a motivating factor in relation to mHealth tracking, there are gender differences in self-monitoring drivers. Second, it demonstrates how establishing new spaces for the implementation of health regimes using fitness apps can improve collegiality and social capital among sports fans.

There are, however, still unanswered questions raised by these data, which should be investigated further. First, the long-term viability of self-tracking commitments is not yet guaranteed. After more than a decade of people downloading mobile health apps, there is still a rapid turnover of apps and their perceived utility changes over time. One of the major challenges with effectively deploying such technology is maintaining relevance for a community to provide ongoing motivation to be active. Using the power of community fandom and this connectivity through mHealth applications can help with this.

Further research should investigate the growing expansion of the data gaze, as well as any perceived dissatisfaction with the imaginary data [42] by building upon previous work [6,7]. As more people become concerned about the power of algorithms and what organisations do with their data, there may be a similar reluctance to constantly track one’s behaviour for fear of succumbing to the way it may skew our experience of the world. The maintenance of credibility and how the design interface allows users to have confidence in how their data are monetised, as well as its informational integrity, is central to this concern. If users conclude that their continued use of the app does not result in long-term changes for the better, they may be less likely to continue using it. Future research could also further explore our key themes to unpack them into a number of sub-themes.

Finally, as fans are encouraged to engage in self-tracking for health, key questions about what types of behaviours and under what conditions could support a more agile and personalised health approach is being raised. This was especially important given that sports fans were forced to avoid stadiums during the COVID-19 pandemic. While elite sports clubs rushed to find ways to entice their fans back into the stadium through experimental media platforms, self-tracking apps may be a more effective way to maintain club loyalty and engagement while also contributing to their community’s health, wellbeing and social capital.

## Figures and Tables

**Figure A1:**
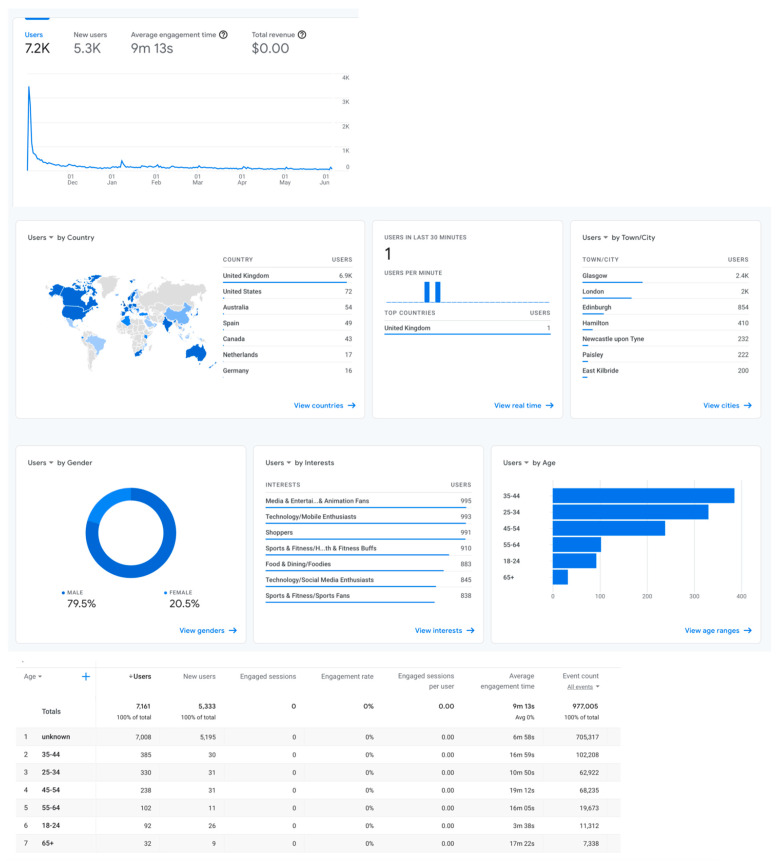
Fitbears Google Analytics between 3 November 2019 and 3 June 2020.
